# Systematizing the risk management process in clinical radiotherapy practice: Recommendations of the working group on risk management of the DGMP

**DOI:** 10.1016/j.zemedi.2024.11.001

**Published:** 2024-11-22

**Authors:** Dominik Kornek, Cordelia Hoinkis, Natasa Milickovic, Ailine Lange, Alena Knak, Manuel März, Mieke L. Möller, Markus Buchgeister

**Affiliations:** a Department of Radiation Oncology, Universitätsklinikum Erlangen, Friedrich-Alexander-University Erlangen-Nürnberg (FAU), 91054 Erlangen, Germany; b Comprehensive Cancer Center Erlangen-EMN (CCC ER-EMN), 91054 Erlangen, Germany; c Department of Radiotherapy and Radiation Oncology, Faculty of Medicine University Hospital Carl Gustav Carus, Technische Universität Dresden (TUD), 01307 Dresden, Germany; d Department of Medical Physics and Engineering, Radiation Oncology Clinic, Sana Klinikum Offenbach, 63069 Offenbach, Germany; e MVZ Prüner Gang, 24103 Kiel, Germany; f Klinik für Strahlentherapie, Klinikum Herford, 32049 Herford, Germany; g Department of Radiotherapy, Regensburg University Medical Center, 93053 Regensburg, Germany; h Radiologische Allianz Hamburg, 20357 Hamburg, Germany; i Faculty of Mathematics-Physics-Chemistry (II), Berliner Hochschule für Technik (BHT), 13353 Berlin, Germany

**Keywords:** Risk management, FMEA, Radiotherapy, Best practice, Patient safety

## Abstract

**Purpose:**

The *Deutsche Gesellschaft für Medizinische Physik* [German Society of Medical Physics] has recently published two coherent reports, No. 25 and No. 28, detailing the design and implementation of a risk management (RM) process for German radiotherapy (RT) departments. This study offers an overview and background of the efforts behind these reports.

**Methods and Materials:**

For three years, up to nine medical physicists (MPs) with practical RM experience held weekly meetings to develop recommendations for a clinical RM process. Care was taken to ensure that the recommendations were equally applicable to RT departments of various sizes. A process-based method derived from the failure mode and effects analysis (FMEA) was created to identify and address risks from unintentional radiation exposure. This method was applied to exemplarily analyze the hazardous scenarios in breast RT using surface guidance and deep inspiration breath hold (DIBH) techniques. Three common criticality methods—risk matrix, risk priority number, and action priority—were applied, and each step was schematically explained for first-time users. Each report was peer-reviewed by two radiation oncologists and 11 MPs.

**Results:**

In report No. 25, basic requirements were outlined for running the RM process, conducting risk assessments, and monitoring clinical procedures. A three-year plan-do-check-act cycle was proposed for continuous improvement. In report No. 28, general process lists for external beam radiotherapy (EBRT), brachytherapy, and radionuclide therapy were designed. Based on the EBRT process list, 45 hazardous scenarios in the surface-guided breast RT in DIBH were identified. Two scenarios were used to illustrate handling instructions for the three criticality methods.

**Conclusions:**

The recommendations provide clinical MPs and other health professionals with a pragmatic approach to RM, balancing both the needs of smaller practices and larger clinics in Germany. The risk of unintended exposures of patients is viewed acceptable once it has been lowered to a state that is as low as reasonably achievable.

## Introduction

Radiotherapy (RT) plays an essential role in cancer care alongside surgery, chemotherapy, and immunotherapy. Radiation is applied in various forms, most commonly through external beam radiotherapy (EBRT), where high doses of ionizing radiation are delivered conformally to tumorous tissue. A multidisciplinary team consisting of radiation oncologists (ROs), medical physicists (MPs), dosimetrists, radiation therapists, and nurses administers the treatments. However, as with any medical procedure, there is an inherent risk of treatment errors. Despite errors in RT are very rare [1], further actions should be taken to keep this risk as low as possible. To enhance patient safety, prospective risk assessment, supplementing the more common retrospective incident analysis, has been recommended by several organizations [1], [2] and ultimately mandated by the EU directive 2013/59/EURATOM.

The *Strahlenschutzverordnung* [Radiation Protection Ordinance] enforces prospective risk assessment in section 126. However, while the *what* is clearly defined, the *how* remains open. Therefore, questions about the risk assessment method, the risk acceptance, the involved staff, or the appropriate level of detail vary among RT departments and competent authorities. As most RT departments had rated their knowledge of risk management (RM) as “satisfactory” or worse in a survey, it is not surprising that a need for more RM guidelines was expressed [3]. What is clear is the w*ho*, as according to section 132 of the *Strahlenschutzverordnung*, MPs are tasked with performing risk assessments.

In response to *how* MPs should conduct risk assessments, the *Deutsche Gesellschaft für Medizinische Physik* [German Society of Medical Physics] (*DGMP*) founded a working group on RM in 2017. Annual meetings followed, and in the meeting of 2021, a dedicated working committee was formed to address the need for more RM guidelines. Over three years, the committee authored two coherent reports.

*DGMP* report No. 25 [4] recommends basic requirements about the outer framework within which the actual risk assessment takes place—the RM process. It is mainly directed to the *Strahlenschutzverantwortlichen* [radiation protection executive] who should ensure that these requirements are met. *DGMP* report No. 28 [5] is mainly dedicated to the risk analysts and provides practical demonstrations of the concepts described in report No. 25. In this study, the process of the development of these recommendations is presented, thereby allowing other states, especially EU members who are also affected by the EU directive 2013/59/EURATOM, to assess the current state of prospective risk assessment in Germany.

## Methods and materials

The *DGMP* working group on RM established a dedicated working committee in March 2021, consisting initially of six honorary MPs experienced in clinical RM. In the committee’s first meeting, it was decided to lay the foundations of a structured RM process, which, while not formally required by the *Strahlenschutzverordnung*, was deemed necessary for systematic risk assessments. This is because, prior to performing risk assessments, the objectives, the methods, the risk level categorizations and corresponding acceptance criteria should be addressed. The RM process also deals with formal questions regarding resources, documentation, verification, and improvement.

The decision to define basic RM process requirements in a first step led to defining risk assessment guidelines in a second step. As it was important to inform the German community as soon as possible, these two steps were processed in two subsequent reports.

A group of two executive MPs, three clinical MPs and an RM consultant with a background in medical physics authored report No. 25. Five of the six members represented three university hospitals, one hospital, and one medical practice. Based on the ISO 31000 (*Risk management – Guidelines*) and ISO 14971 (*Medical devices – Application of risk management to medical devices*) norms, the model RM process was adapted to clinical RT processes. The stipulations of the *Strahlenschutzverordnung* and the joint recommendations [6] of the *Bundesamt für Strahlenschutz* [Federal Office for Radiation Protection], *Deutsche Gesellschaft für Radioonkologie* [German Society for Radiation Oncology], *DGMP*, and *Deutsche Gesellschaft für Nuklearmedizin* [German Society for Nuclear Medicine] were considered. The draft report was then presented to the participants of the working group’s annual meeting in March 2022 for feedback. The finalized report was subsequently reviewed by two ROs and 11 MPs before publication.

A group consisting of two executive MPs, five clinical MPs and a professor of medical radiation physics composed the report No. 28. These members represented three university hospitals, two hospitals and two medical practices. A method based on the failure mode and effects analysis (FMEA) was developed. The FMEA was chosen due to the broad popularity (see, e. g., references [7], [8], [9], [10], [11], [12], [13], [14]) of this method—a result of the widespread impact of the joint recommendations [6] and the report of task group 100 of the American Association of Physicists in Medicine (AAPM) [10]. The FMEA is a team-oriented and analytical approach intended to evaluate risks of failure within a process, analyze the associated failure causes and effects, and identify controls to mitigate these risks [15]. The report incorporated three different FMEA criticality methods—risk matrix, risk priority number (RPN), and action priority (AP)—to address more RT departments. General process lists, applicable to typical German settings, were compiled for EBRT, brachytherapy, and radionuclide therapy. Both the EBRT and brachytherapy process maps were based on the AAPM’s process maps [16] and the radionuclide therapy process map was created by the *DGMP* working group on RM. Lastly, an exemplary FMEA using the risk matrix, RPN, and AP was performed for the surface-guided breast RT in deep inspiration breath hold (DIBH). The general EBRT process list was used to identify the changes introduced by the breast RT workflow which then were analyzed in an illustrative step-by-step manner. Again, the draft report was presented at the annual meetings of the working group in March 2023 and 2024 and then reviewed by two ROs and 11 MPs before publication.

## Results

After a period of three years, both the *DGMP* reports No. 25 and No. 28 have been made available as online resources [4], [5]. Below, the core recommendations from these reports are provided.

### Core recommendations for the RM process

**Figure 1:**
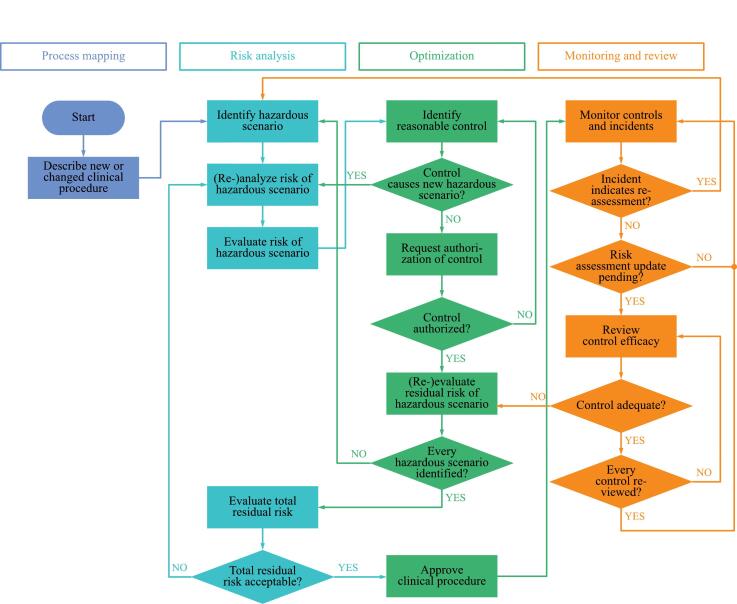
Flowchart of the risk management process initiated through the introduction of a new or changed clinical procedure. Risks are analyzed, evaluated, and optimized before the clinical procedure is approved, launched, and monitored.

The recommended RM process includes elements from process mapping, risk assessment, optimization, and monitoring and review, as shown in Figure 1. This systematic process aims to reduce the total residual risk of clinical procedures to a state that is as low as reasonably achievable (ALARA). The top management may authorize the identified risk mitigation strategies based on economic, social or other factors. However, rejected controls require alternative solutions to be identified. If no more reasonable controls can be identified, then the associated risks need to be documented as residual risks. Once all controls are implemented, the clinical procedure is approved, monitored, and continuously optimized to further improve its safety.

The RM process should be documented through a standard operating procedure (SOP). This SOP should detail necessary qualifications, roles, and competencies of staff as well as time slots, facilities, and tools to perform risk assessments. The methodology of prospective risk assessments should be fully described, i.e., the specific risk assessment method (e.g., FMEA), the risk rating system (e.g., a five-step system for the occurrence and severity of risks), the criticality method (e.g., risk matrix), the risk acceptance criteria (e.g., “acceptable”, “tolerable”, “not acceptable”), and the multidisciplinary team that performs the risk assessments. The lower and upper limits of the risk rating system should be appropriate for the RT department’s clinical procedures. This SOP forms the basis for specific RM plans for each clinical procedure. The RM plan defines the scope, activities, and results of the risk assessment which can then be reviewed post-assessment.

The importance of thorough documentation throughout the RM process is met by creating an RM file (see Table 1). The RM plan as well as the RM report should be part of the RM file. Furthermore, all RM activities should be documented in the RM file.

**Table 1:** Structure of the risk management (RM) file used for documentation of all activities related to the risk assessment of clinical procedures.

1.	RM plan
	a. Introduction
	b. Scope of planned RM activities
	c. Responsibilities and authorities
	d. Criteria for risk analysis
	i. Categorization of the severity (*S)* of harm resulting from a hazardous scenario
	ii. Categorization of the probability of occurrence (*O)* of a hazardous scenario
	iii. Categorization of the probability of detectability (*D)* of a hazardous scenario
	iv. Categorization of the probability of net occurrence (*NO)* of a hazardous scenario resulting in harm
	e. Criteria for risk acceptance
	f. Review of RM activities
	g. Method for evaluating the total residual risk resulting from all hazardous scenarios
2.	Records of activities of the risk assessment team
	a. Identification of hazards and hazardous scenarios
	b. Analysis and evaluation of each hazardous scenario
	c. Prioritization of hazardous scenarios to deduce controls
	d. Identification of controls and evaluation of residual risk for each hazardous scenario
	e. Evaluation of risks arising from controls
	f. Evaluation of the total residual risk
3.	RM report
	a. Introduction
	b. Review of the RM plan
	c. Executive summary of the total residual risk
	d. Approval of the new or changed clinical procedure
	e. Determination of periodic review of the risk assessment and implementation of identified controls

### Core recommendations for the risk assessment

#### Process mapping

As depicted in Figure 1, the first step of the RM process is to describe the clinical procedure on a main and sub-step basis, which could be tabularly or graphically. In a reasonable approach, the RT department’s standard clinical procedure should be defined so that procedural changes can be clearly distinguished and assessed. In *DGMP* report No. 28, general process lists for EBRT, brachytherapy, and radionuclide therapy, containing 88, 81, and 33 process sub-steps, respectively, have been proposed as standard clinical procedures for German RT departments. These lists were recommended as starting points for process maps. In the exemplary surface-guided breast RT in DIBH, 15 out of 88 process sub-steps were considered relevant.

#### Risk analysis

**Figure 2:**
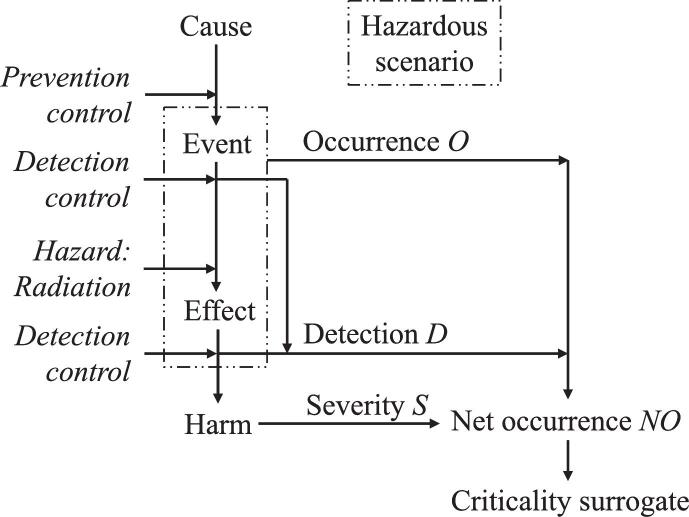
Sequence of a hazardous scenario, its initiating cause and resulting potential harm, with parameters for determining criticality (occurrence O, detectability D, net occurrence NO, severity S).

For each process step, all foreseeable hazardous scenarios, causes, and potential harms should be identified. Here, a hazardous scenario was modeled by an initiating event and an immediate effect, as shown by the dash-dotted box in Figure 2. In all hazardous scenarios, the underlying hazard should be ionizing radiation. To identify hazardous scenarios, brainstorming for events that are caused by incorrect prescriptions, incorrect executions of the prescription or poor time management was recommended. Moreover, available information such as vendor information or known incidents should be considered. Such incidents can be found in public incident reporting systems, some of which are given in Table 2. Table 3 lists 16 common events identified through learning from 625 incidents that occurred in the department of one of the authors over a period of about four years. It was advised to consider these initiating events when identifying hazardous scenarios. Using this approach, 45 hazardous scenarios could be identified for the 15 process steps of the surface-guided breast RT in DIBH.

**Table 2:** Public incident reporting systems relevant to radiotherapy.

**Incident reporting systems**	**Location**
BeVoMed – Short notices†	https://www.bfs.de/DE/_bevomed/_strahlentherapie-kurz-info/kurz-info.html
BeVoMed – Annual reports	https://www.bfs.de/DE/themen/ion/anwendung-medizin/bevomed/jahresbericht/jahresbericht_node.html
BfArM – DMIDS	https://www.bfarm.de/DE/Medizinprodukte/Portale/DMIDS/_node.html
BfArM – Field safety notices	https://www.bfarm.de/DE/Medizinprodukte/Aufgaben/Risikobewertung-und-Forschung/Massnahmen-von-Herstellern/_node.html
MAUDE	https://www.accessdata.fda.gov/scripts/cdrh/cfdocs/cfmaude/search.cfm
NYPORTS †	https://www.health.ny.gov/facilities/hospital/nyports/
Public Health England	https://www.gov.uk/government/publications/radiotherapy-errors-and-near-misses-data-report
RIRAS	https://www.cars-pso.org/
RO-ILS †	https://www.astro.org/Patient-Care-and-Research/Patient-Safety/RO-ILS
ROSEIS †	https://roseis.estro.org/
SAFRON †	https://rpop.iaea.org/SAFRON/

† user credentials required.

**Table 3:** 16 events potentially contributing to hazardous scenarios during (external beam) radiotherapy (RT) processes.

**Main process**	**Events**
Physician–patient consultation and physical examination	RT (dose level and/or target volume) incorrectly prescribed
	Previous RT treatments not adequately considered
	Pacemaker, spinal cord stimulator, etc. not adequately considered
Medical treatment planning	Target volume(s) not contoured according to the prescription
	Medical history, tumor board decisions, imaging studies, or lab results not adequately considered
Physical treatment planning	Physical treatment planning incorrectly executed (e.g., incorrect transfer of prescribed fraction dose and total dose, poor dose conformity, etc.)
	Data from treatment planning system incorrectly transferred
	Incorrect plan approved for treatment
	Set-up instructions for image guidance incorrectly planned
Day 1 treatment, day *N* treatment, on-treatment quality management	Patient incorrectly positioned or immobilized
	Adaptive plan changes incorrectly executed
	Combined radio-chemotherapy incorrectly executed
General	Patient incorrectly identified
	Information inadequately communicated or documented
	IT failure or disruptions
Emergency treatment	Emergency RT inadequately executed

For the risk analysis of the hazardous scenarios, the four following parameters were suggested (see Figure 2) to determine a criticality surrogate:

• Severity (*S*), categorizing the harm due to the hazardous scenario.
• Occurrence (*O*), categorizing the frequency of the hazardous scenario.
• Detectability (*D*), categorizing the probability to detect the hazardous scenario (initiating event *or* immediate effect) before the patient comes to harm.
• Net occurrence (*NO*), categorizing the frequency of the hazardous scenario resulting in harm (combination of occurrence and non-detectability).

For the risk matrix, *S* and *NO* were suggested and sub-divided into five categories each, resulting in a criticality matrix with 25 cells. For the RPN and the AP, the three parameters *S*, *O*, and *D* were suggested and sub-divided into ten categories each. The proposed risk rating system is given in Table 4.

**Table 4:** Five-step and ten-step risk rating systems for the risk matrix, and the risk priority number (RPN) and the action priority (AP), respectively.

**Categories**		**Severity** **S**	**Occurrence** **O**	**Detectability** **D**	**Net occurrence** **NO**
**for the risk matrix**	**for the RPN and AP**				
I	1	Negligible: no harm induced	Very low: much less frequent than yearly	Certain: detected within the same process sub-step	Very rare
II	2-3	Low: minor therapy delay, small dosimetric or geometric deviation	Low: less frequent than yearly	High: detected in subsequent process sub-step or through verification by two people	Rare
III	4-6	Medium: Unexpected, but moderate harm due to larger dosimetric or geometric deviations	Medium: yearly	Medium: Verification by one person	Occasional
IV	7-8	High: Severe harm	High: monthly	Low: Not systematically verified	Frequent
V	9-10	Very high: Life-threating harm or death	Very high: weekly or more frequent	Very low: not verified	Very frequent

#### Optimization

A two-step optimization process was recommended, consisting of a pre-screening and screening step. In the pre-screening step, all hazardous scenarios should be sorted in descending order of the criticality surrogate. For example, using the RPN, the list should go from highest to lowest RPN. Then, in the screening step, the sorted hazardous scenarios should be evaluated in terms of risk classes, using the risk acceptance criteria defined in the RM plan. The risk class was recommended to be the key decision criterion for developing further controls.

Controls should be identified and risks re-analyzed until acceptable or tolerable risk classes can be assigned, provided the risks are reduced to a state that is ALARA. Once all to-be-implemented controls have been identified, the sequence of their implementation should be determined under consideration of costs and efforts.

Table 5 illustrates the results obtained from process mapping through risk assessment to optimization for an exemplary hazardous scenario of the surface-guided breast RT in DIBH (the underlying risk acceptance criteria and reasoning can be found in detail in report No. 28 [5]).

**Table 5:** RM activities for an exemplary hazardous scenario potentially occurring during the surface-guided breast RT in DIBH under application of the risk matrix, risk priority number (RPN) and action priority (AP) methods. SGRT: surface guided radiotherapy. RTT: radiation therapist. MP: medical physicist. ALARA: as low as reasonably achievable.

**Activity of risk analysis**	**Sub-activity of risk analysis**	**Subject of analysis**	**Risk matrix**		**Risk priority number**		**Action priority**	
			***S-NO***	**Risk level**	***S-O-D***	**RPN**	***S-O-D***	**AP**
Process mapping	Main process delineation	Main process	Treatment delivery (day 1 treatment)					
	Sub process delineation	Sub-process	Utilization of motion management & beam trigger					
Risk assessment	Risk identification	Initiating event of hazardous scenario	Override of interlock after incorrect reference surface (e. g., obsolete reference surface, reference surface of another patient, etc.) has been uploaded to the SGRT system					
		Immediate effect of hazardous scenario	Wrong volume irradiated during first fraction					
		Causes	Inattention; insufficient training; lack of standardized procedures					
		Current prevention controls	n/a					
		Current detection controls	Manual check by RTT if uploaded reference surface is correct; subsequent imaging (cone beam computed tomography or portals) before beam delivery					
	Risk analysis	Criticality	III-II	R11	5-2-3	30	5-2-3	Low
	Risk evaluation	Risk class	Tolerable		Tolerable		Action recommended	
Optimization	Identification of controls	Recommended prevention controls	n/a					
		Recommended detection controls	Override of interlock only after careful review and under application of the “four eyes principle” (RTT & MP)					
Risk re-assessment	Risk re-analysis	Criticality	III-I	R6	5-2-2	20	5-2-2	Low
	Risk re-evaluation	Risk class	Acceptable (ALARA)		Acceptable (ALARA)		No further action needed (ALARA)	

#### Monitoring and review

Once the clinical procedure has been approved for clinical use, a monitoring and review process should follow. As can be seen in Figure 1, the monitoring and review process is a closed loop acting as a plan-do-check-act cycle of continuous improvement. In this process, the estimations about both the risks and effectiveness of controls should be verified. The verifications should then be used as input for risk assessments updates.

## Discussion

This work presents an overview of the development of core recommendations of the *DGMP* working group on RM.

In *DGMP* report No. 25, an RM process for performing risk assessments was outlined, modeled after ISO 14971 due to the similarity of the required tasks. Relevant legal tasks are defined in sections 105, 108, 109, and 126 of the *Strahlenschutzverordnung*, which cover preparatory measures, incident reporting, incident investigation, and risk assessments, respectively. By adapting the main concepts of ISO 14971 to clinical procedures, it was found that the aforementioned sections could be met. The RM process also agrees in large parts with concepts from ISO 31000 and recommendations from international organizations. For example, while section 132 of the *Strahlenschutzverordnung* describes risk assessment as a key area of involvement for MPs, many organizations [1], [17], [18], [19] advocate for a multidisciplinary team approach. In highly complex clinical procedures requiring input from numerous individuals [1], having only the MP perform the risk assessment may lead to unilateral results. A multidisciplinary team approach prevents this.

Although the *Strahlenschutzverordnung* does not formally require an RM process, its concepts have proven useful in the experience of the authors. In the department of one of the authors, for example, the risk assessment method was being adapted progressively while performing initial risk assessments, often questioning its validity, for instance, concerning the risk rating criteria or criticality methods. Such foreseeable challenges (as a specific example, the known problems of the RPN [20], [21], [22] that new analysts come across and do not yet know of) may render team meetings cumbersome and potentially ineffective but could be avoided by fully clarifying these issues in the RM process SOP beforehand.

*DGMP* report No. 28 proposed a process-oriented modified FMEA method for performing risk assessments. Process-oriented risk assessments were found reasonable for RT departments to analyze risks arising from human-human and human-system interactions in their clinical procedure design. The main difference from standard FMEA lies in the substitution of *failure modes* with *hazardous scenarios*. Failure modes represent any manner of failure, not all of which affect radiation-related patient safety, whereas hazardous scenarios are limited to those where radiation causes harm. Hazardous scenarios can be identified through brainstorming and learning from incidents. It should be noted here that this approach greatly focuses on observed risks. If there is no experience or information available about a certain procedure, then conventional FMEA, statistical process control [10], preliminary hazard analysis [1] or system theoretic process analysis [23] might be more suitable approaches.

Another difference from standard FMEA is sorting identified risk mitigation controls. Sorting hazardous scenarios by any criticality method (e.g., the RPN) suggests implementing controls in the same order, i.e., controlling the highest-risk hazardous scenarios first. However, as Cox [24] illustrated (originally for risk matrices), criticality methods often lack considerations of control effectiveness or costs. Therefore, from an economic perspective, where the interaction of all controls needs to be optimized under a given budget to minimize the total residual risk (i.e., ALARA), risk-sorted lists may lead to suboptimal resource allocations [24]. In order that the multidisciplinary RM team is enabled to optimize the choice of controls, appropriate budgets balanced against the risk of the clinical procedure should be communicated to the team pre-assessment.

In Table 5, the fact that all three criticality methods recommended another control to make the risk ALARA might create the impression that they always result in the same action. This is not true, as the methods are not interchangeable. For instance, if the AP is defined as “high” for an *S-O-D* combination of 8-3-3 and “medium” for 3-3-8, the RPN (72) would be the same in both cases. Therefore, RT departments should be aware of each method’s advantages and disadvantages when selecting one. It can also be seen that an additional detection control was recommended despite the initial risk already being low (e. g., the initial AP was rated “low”). This is because risk acceptance criteria (e. g., “acceptable” if the AP is “low” or if the RPN is less than 30) are, after all, arbitrary [20] in contrast to the ALARA principle.

In conclusion, we addressed the urgent need for more pertinent information about RM [3], particularly prospective risk assessments, and the reports should be consulted for practical guidance on performing these assessments for the first time. The recommendations were designed to be as simple as possible while still extensive enough to implement a reasonable RM process with limited resources, such as time and staff. These are *minimum* viable recommendations, and a more comprehensive RM process should be encouraged whenever possible. For example, the AAPM TG-100 [10] combined FMEA with fault tree analysis, another method that identifies the reliability of a clinical procedure in the event of simultaneous failures which revealed missing redundancies and resulted in a more comprehensive risk profile. However, the *DGMP* reports focused on producing fast and effective results for initial risk assessments under section 126 of the *Strahlenschutzverordnung*.

Ensuring adherence to RM requirements in RT departments remains an ongoing challenge, as there are currently no specific legal regulations on how RM should be implemented. While the reports were primarily intended for RT departments, competent authorities and *Ärztliche Stellen* [medical bodies] could also benefit from using them. As Malicki et al. recommended, European national authorities and other regulatory bodies overseeing patient safety in RT should integrate an RT-specific RM system into the certification processes of healthcare organizations [25]. Competent authorities, for example, could establish minimum expectations for risk assessments and reference both reports when demanding risk assessments. Similarly, *Ärztliche Stellen* could design standardized criteria for clinical audits to increase the overall impact of RM. This could give clinical MPs and other health professionals the guidance they seek from their respective societies [3]. However, it is important that the reports are not seen as prescriptive or restrictive. They offer only one of many possible approaches to effective RM, and individual RT departments should retain flexibility in how they apply RM principles.

## CRediT authorship contribution statement

**Dominik Kornek:** Writing – original draft, Visualization, Methodology, Conceptualization. **Cordelia Hoinkis:** Writing – review & editing, Methodology, Conceptualization. **Natasa Milickovic:** Writing – review & editing, Methodology, Conceptualization. **Ailine Lange:** Writing – review & editing, Methodology, Conceptualization. **Alena Knak:** Writing – review & editing, Methodology, Conceptualization. **Manuel März:** Writing – review & editing, Methodology, Conceptualization. **Mieke L. Möller:** Writing – review & editing, Methodology, Conceptualization. **Markus Buchgeister:** Writing – review & editing, Supervision, Methodology, Conceptualization.

## Declaration of competing interest

The authors declare the following financial interests/personal relationships which may be considered as potential competing interests: The authors declare that they have no known competing financial interests or personal relationships that could have appeared to influence the work reported in this paper. The Universitätsklinikum Erlangen and IBA Dosimetry GmbH (Schwarzenbruck, Germany) are the funding recipients of the grants mentioned in the Acknowledgments. Neither the StMWi nor IBA Dosimetry GmbH had any involvement with the work reported in this paper.
